# The impact of pediatric blood and marrow transplant on parents: introduction of the parent impact scale

**DOI:** 10.1186/s12955-015-0240-6

**Published:** 2015-04-09

**Authors:** Katherine E Heinze, Angie Mae Rodday, Marie T Nolan, Kristin Bingen, Mary Jo Kupst, Sunita K Patel, Karen Syrjala, Lynnette Harris, Christopher Recklitis, Lisa Schwartz, Stella Davies, Eva C Guinan, Robert Noll, Grace Chang, Susan K Parsons

**Affiliations:** Johns Hopkins University School of Nursing, 525 N Wolfe Street, Baltimore, MD 21205 USA; Tufts Medical Center, 800 Washington Street #345, Boston, MA 02111 USA; Medical College of Wisconsin, 8701 Watertown Plank Road, Milwaukee, WI 53226 USA; City of Hope, 1500 E. Duarte Road, Duarte, CA 91010 USA; Fred Hutchinson Cancer Research Center, 1100 Fairview Avenue N, Seattle, WA 98109 USA; Baylor College of Medicine, One Baylor Plaza, Baylor, TX 77030 USA; Dana-Farber Cancer Institute, 450 Brookline Avenue, Boston, MA 02215 USA; University of Pennsylvania School of Medicine, 3501 Civic Center Boulevard CTRB 10311, Philadelphia, PA 19104 USA; Cincinnati Children’s Hospital Medical Center, 3333 Burnett Avenue, Cincinnati, OH 45229 USA; Children’s Hospital of Pittsburgh of PUMC, 4401 Penn Avenue, Pittsburgh, PA 15224 USA; Department of Psychiatry, Harvard Medical School, Boston, MA 02115 USA

**Keywords:** Blood and marrow transplantation, Caregiving, Stress, Parent impact

## Abstract

**Background:**

Parents often experience stress-related complications when their child requires blood and marrow transplant (BMT). Previous studies have described the emotional toll BMT places on parents during the acute phase of care and within the context of clinical complications. In this paper we introduce the Parent Impact Scale (PARimpact), designed to capture physical and emotional challenges of the child’s health on the parent. The primary aim of this paper is to examine psychometric properties of PARimpact, and the secondary aim is to explore factors associated with PARimpact scores for further hypothesis generation.

**Methods:**

This analysis used a merged dataset of two longitudinal studies. Accompanying parents (n = 363) of children undergoing BMT were surveyed up to six times from pre-BMT baseline to one year after their child’s BMT. For this analysis, pre-BMT baseline responses to PARimpact were used to examine the factor structure with Principal Component Analysis (PCA) and Exploratory Factor Analysis (EFA). Construct validity was assessed, and multivariable regression was used to examine relationships between PARimpact and BMT clinical variables.

**Results:**

PCA and EFA revealed a one-factor solution with acceptable item loading; Cronbach’s α was 0.83 at baseline. Hypothesized differences in known groups were detected for BMT complications with significantly higher PARimpact scores for those with vs. without each complication. In the adjusted multivariable regression models, acute graft versus host disease (b = 5.3; p = 0.03), end organ toxicity (b = 5.9; p < 0.01), and systemic infection (b = 9.1; p < 0.01) were associated with significantly higher mean PARimpact scores in the first 3 months following transplant. After the first 3 months to 1 year post BMT, systemic infection was associated with increased mean PARimpact scores (b = 19.2; p < 0.01).

**Conclusions:**

Initial results suggest that the PARimpact is valid and reliable. Our finding that clinical complications increase the impact of BMT on the caretaking parent indicates the need for BMT healthcare professionals to identify these events and help parents navigate the BMT course. Clinical application of the PARimpact scale should be considered to identify high-risk families and provide targeted interventions to augment care.

## Introduction

Blood and marrow transplant (BMT) offers children with life-threatening illness, and their families, hope of durable cure when no other curative treatment is available [1]. However, parents of children undergoing BMT often experience a great deal of stress [2], and are at risk for short and long-term psychosocial sequelae, such as depression, anxiety, post-traumatic stress symptoms, and decreased quality of life (QOL) [3-6]. While some prospective studies have shown that parental well-being is most affected during the acute phase of the BMT and then improves with time, there is evidence that the pattern is more nuanced, and is related to other variables, such as previous emotional history and the clinical complications of the BMT [5,7-10]. Furthermore, retrospective studies show some bereaved and non-bereaved parents continue to experience distress for years after the BMT [11-13].

Enhancing our understanding of the parent, family, and BMT characteristics that are associated with increased parental impact may help clinicians identify parents most at risk, and lead to the development of interventions to support parents through the BMT trajectory. In one study of 49 parents/guardians of BMT recipients, 81% of parents/guardians reported they felt proceeding to BMT was their only choice, given its life-saving potential [14]. Because of the high stakes and demands of the treatment, support for these potentially vulnerable parent caregivers is paramount.

Previous research has documented that parental distress [9] and diminished parent emotional functioning are associated with BMT [7]. What has not been described is the direct effect of the child’s emotional and physical condition on parents, and their ability to manage other obligations, such as work. In this paper we introduce the Parent Impact Scale (PARimpact), a scale within the Child Health Ratings Inventories (CHRIs), [15] designed to capture the physical and emotional impact of the child’s health condition on the parent. In this context, ‘impact’ is considered in the negative sense, conferring challenges to the parent. The primary aim of this analysis is to examine the psychometric properties of the PARimpact as a “stand-alone” scale of the impact of children’s BMTs on parents, and parents’ ability to meet their own needs. A secondary aim is to explore factors associated with PARimpact scores for further hypothesis generation.

## Methods

Data for this analysis were drawn from a merged dataset of two completed dyadic longitudinal health-related quality of life (HRQL) studies comprised of children undergoing BMT and their accompanying parent (Journeys to Recovery (JTR) and HSCT-CHESS™, described elsewhere [16-18]). Data were collected at eight pediatric BMT centers across the United States from 2003–2011 at clinically relevant time points: pre-BMT baseline, 45 days (represents the end of the inpatient hospitalization period), 3 months (end of the acute BMT period), 6 months, 9 months (HSCT-CHESS™ only), and one year after transplant (designed to capture the late complications and recovery period). Both source studies, including data from the PARimpact Scale described in this paper, were approved by the Tufts Medical Center Institutional Review Board (IRB) and the IRB at each research center. IRB approval was also obtained from Tufts Medical Center and Johns Hopkins University to combine the data for this analysis in accordance with the Helsinki Declaration.

Survey data in this study were drawn from the CHRIs-General, a generic HRQL measure with child, adolescent, and parent versions. The CHRIs contains scales to measure HRQL and related constructs: HRQL scales measure emotional, physical, and role functioning, which together represent the multi-dimensional construct of HRQL [19], while the PARimpact and global QOL scales measure related constructs. The separate global QOL scale consists of nine items in which the respondent rates their overall quality of life in the areas of physical health, emotional health, and social health [15]. The CHRIs scales have been extensively validated within the pediatric BMT population [6,15].

### Study sample

Accompanying parents of pediatric patients aged 2 months to 18 years who were scheduled to undergo BMT at study sites were consecutively recruited. Dyadic participation of the parent and child was required. Inclusion criteria for parents were: ability to speak/understand English, at least 18 years old, parent or legal guardian of the BMT patient, and ability to provide consent to participate both for him or herself as well as for the BMT patient. While only parent data were included in this analysis, age-eligible child participants also provided assent (participants < 18 years) or consent (participants ≥ 18 years) for his or her participation. Overall, 62% of eligible dyads consented to participate. Common reasons for non-participation included child refusal (27%), parent refusal (44%), and medical reasons (3%). Participants did not differ from non-participants based on child age, gender, or race/ethnicity.

A total of 423 parents were enrolled in the JTR and HSCT-CHESS™ studies; 60 parents of children less than 5 years old were excluded from this analysis because in the JTR study parents of children in this age group did not complete the CHRIs, which left 363 parents in this analysis.

### Measures

#### Parent Impact Scale

The PARimpact was developed as a separate scale within the parent-proxy version of the CHRIs-General to measure the response of the parent to the child’s physical and emotional health needs. The scale consists of four items with five response options on a frequency-based Likert-type scale ranging from “none of the time” to “all of the time” (see Table 1 for PARimpact questions). The scale scores range from 0–100 with higher scores indicating greater parent impact. Starting 45 days after transplant (“day 45” the second data collection point), three additional open-response items were administered alongside the PARimpact scale items to ascertain the actions that parents took in the previous seven days as a result of their child’s BMT. These included the number of missed work days in the past week due to the child’s health, missed work days due to the parent’s health, and the number of days someone came to help the family in the past week. While these items were not designed to be part of the scored PARimpact Scale, they are a related set of items that complement the PARimpact scale. Among these three items we focused on missed workdays due to the child’s health for this analysis.

**Table 1:** **PARimpact items and scores**

**Pre-BMT baseline scores (Cronbach’s α = 0.83)**					
	**n**	**Mean**	**Std. Dev.**	**% Floor**	**% Ceiling**
Summary score*	363	51.41	24.65	2	4
Item summaries					
Child’s physical health causes suffering	363	60.54	28.32	6	19
Child’s physical health limits time for parental needs	363	50.96	31.02	15	12
Child’s emotional health causes suffering	363	51.24	31.35	13	15
Child’s emotional health limits time for parental needs	363	42.91	30.70	20	8

*PARimpact is scored from 0–100 with higher scores indicating greater impact.

#### Parent Emotional Functioning

The Parent Emotional Functioning scale within the parent-proxy CHRIs General consists of seven questions measuring parent mood, anxiety, and distress [5,6,20]. The response options for each item utilize a five-point Likert-type scale. Scale scores range from 0–100 with higher scores denoting better emotional functioning. In the JTR study, the Parent Emotional Functioning scale demonstrated strong prediction of DSM-IV Axis 1 disorders, based on area under the receiver operating characteristic curve [21] for predicting a threshold or sub-threshold disorder from related modules of the Structured Clinical Interview for DSM-IV Axis 1 disorders (c-statistic = 0.75) [20].

#### Parent Global QOL

The parent version of the Global QOL scale of the CHRIs consists of nine questions designed to capture the multidimensionality of QOL as physical, mental, and social well-being. Responses are measured with a Likert-type scale with five response options ranging from “poor” to “excellent.” The scale is scored from 0–100, with higher scores indicating higher QOL [16].

#### Parent General Health

The General Health item in the CHRIs is a single summary item of parents’ general health appraisal. Parents were asked, “Overall, how would you rate your health”; response options include a five-point Likert-type scale, which is scored on a 0–100 scale with higher scores indicating better health. This item has been used extensively in clinical practice and research [22] and has been found to be associated with multi-item scales of general health and other markers of disease and clinical outcomes [23].

### Clinical variables

Clinical data were collected at all assessment time points by trained study staff, using standardized data collection instruments. All clinical data were reviewed by the study PI (SKP) for completeness and consistency. Pre-BMT baseline information included time since diagnosis (months), disease category (malignant or non-malignant), and transplant type (related allogeneic, unrelated allogeneic, or autologous). In follow up, specific clinical outcomes variables used in this analysis included early and late BMT complications, defined below.

#### Early BMT complications

We assessed early BMT complications with standardized grading scales that assess both presence or absence and severity of the following: acute graft versus host disease (aGVHD) [24]; end organ toxicity, based on the Bearman Toxicity Scale; [25] and systemic infection, based on the National Cancer Institute’s Common Toxicity Criteria for AE, v. 3.0 [26]. Each of the early BMT complications was dichotomized as follows: grade 2 or higher aGVHD; Bearman Toxicity Score maximum ratings of “intermediate” or “poor” within the first 3 months post-transplant; and grade 3 or higher infection, indicating systemic and/or life-threatening infection.

#### Late BMT complications

Late BMT complications were defined as the extent of chronic graft versus host disease (cGVHD) [27,28] or a systemic infection that occurred after the 3-month data observation [26]. The Bearman scale was not designed for use beyond the 3-month mark post BMT.

#### Demographic variables

Parents’ demographic data, including age, race/ethnicity, education, marital status, household income, work status (e.g., full-time, part-time), and child’s insurance were collected from parent participants at pre-BMT baseline. Parents also reported their child’s race/ethnicity and sex.

### Statistical analysis

Demographic and clinical characteristics were described for the combined study sample using medians (interquartile range [IQR]), means (standard deviations [SD]), frequencies, and percentages at pre-BMT baseline. Parents’ missed work days due to the child’s health were also summarized, and Spearman’s correlation was used to compare this open-response item to the PARimpact scale score from day 45 to 1 year post BMT.

#### Psychometric properties of the PARimpact scale

Principal component analysis (PCA) was used to determine the unidimensionality of the PARimpact scale, based on data collected at pre-BMT baseline. A scree plot was used to retain components with an Eigen value greater than 1.00. Exploratory factor analysis (EFA) was completed to examine factor loadings and uniqueness. Factor loadings >0.4 were considered acceptable [29].

Pre-BMT baseline raw scores, means, SDs, ceiling and floor effects, and percent missing were calculated for each item within the PARimpact. Cronbach’s alpha [30] was calculated to estimate the internal consistency of the scale at pre-BMT baseline. For exploratory scale development, the minimum acceptable Cronbach’s alpha is 0.7, but for established scales the minimum is 0.8 [31].

#### Validation of the PARimpact scale

To assess convergent validity of the PARimpact within the pediatric BMT sample, Pearson correlations were calculated between the PARimpact and other scales of the CHRIs General, including Parent Emotional Functioning, Parent Global QOL, and Parent General Health. Correlations between 0 and .30 were classified as weak, .30-.60 as moderate, and > .60 as strong [32]. We hypothesized that parent impact would be strongly correlated to parents’ emotional functioning, moderately correlated with Global QOL, and weakly correlated with parents’ general health. The correlations were expected to have negative valence due to the directionality of the scales (e.g. higher scores for emotional functioning suggest better functioning, while higher scores for PARimpact suggest more negative impact).

Expected variation of PARimpact scores by known groups was explored using clinically important subgroups, such as early complications, and later complications. For known group comparisons, two separate binary variables were created: (1) Early Complications, which included aGVHD ≥ grade 2 and/or systemic infection and/or “intermediate” or “poor” end organ toxicity; and (2) Late Complications, which included cGVHD and/or systemic infection.

#### Regression analysis

We built two models for regression analysis: an early model and a late model. The early model included data from pre-BMT baseline, day 45, and 3 months. The late model included data after 3 months through 12 months post BMT. In both models, unadjusted longitudinal regressions were completed with demographic and clinical variables using residual maximum likelihood (REML). Timing of repeated assessments was calculated as the number of days since BMT. Based on a likelihood ratio test (data not shown), time was treated as continuous rather than categorical. Variables with an estimated coefficient with p ≤ 0.2 were included in the adjusted multivariable regression model. In the adjusted multivariable longitudinal regressions, variables with p > 0.1 were then removed from the model.

Auto regressive, unstructured, and compound symmetry correlation structures were compared using Akaike information criterion (AIC). To address the possibility that PARimpact scores may have been missing not at random (MNAR) over time, we stratified the final model by the extent and causes of missing data, defining strata as follows: (1) those with missing data due to a medical reason (e.g. child too sick) and (2) those with complete data or those with missing data not due to a medical reason (e.g., logistical reasons, such as transportation or work-related issues). The stratified models (called pattern mixture models, PMM) [33] assume the data are missing at random (MAR) within strata. We compared the stratified to the unstratified model using a likelihood ratio test to assess for the presence of MNAR. SAS version 9.3 was used for all statistical analyses; alpha was set at 0.05.

## Results

### Study sample

Pre-BMT baseline demographic and clinical characteristics are shown in Table 2. The majority of the sample was female and Caucasian with at least some college education. Most parents reported being married or living with a partner (80%), and reported having at least one additional child besides the BMT recipient (84%).

**Table 2:** **Participant characteristics**

	**Mean**		**Standard Dev.**	
Child age	9.6		5.1	
Parent age	38.7		7.5	
Length of illness (months)	11 (Median)		5, 37 (Q1, Q3)	
	**Patient**		**Parent**	
	**n**	**Percent**	**n**	**Percent**
Sex				
Female	169	47	301	83
Male	194	53	62	17
Race				
White	291	81	277	77
Black	24	7	21	6
Asian	11	3	13	4
Other	32	9	47	13
Hispanic ethnicity				
Yes	63	18	64	18
No	297	83	297	82
	**n**		**Percent**	
Parent marital status				
Married/living together	292		80	
Divorced/separated/widowed	49		14	
Never married	18		5	
Other	4		1	
Family income				
<$20,000	31		9	
$20,000-$39,999	89		25	
$40,000-$79,000	114		32	
$80,000 +	123		34	
Child’s insurance				
Private	235		65	
Public	121		33	
None/unknown	7		2	
Parent education				
< High school	21		6	
High school graduate	91		25	
Some college	108		30	
College graduate +	143		39	
Number of siblings				
0	60		17	
1	134		37	
2 or more	169		47	
BMT type				
Autologous	82		23	
Allogeneic – related	89		25	
Allogeneic – unrelated	192		53	
BMT source				
Bone marrow	200		55	
Peripheral blood	126		35	
Umbilical cord blood	35		10	
Other/combined	2		1	
Death of child within 12 months				
Yes	63		17	
No	300		83	
Disease relapse within 12 months				
Yes	48		13	
No	315		87	

### PARimpact scores

Table 1 displays PARimpact scale and item means, SDs, and floor and ceiling effects at pre-BMT baseline. For each item, the full range of possible responses from 0–100 was utilized. Variability across the scale’s items was similar (SD 28.3-31.4). Responses at the floor and ceiling were <5% for the summary score, and ≤20% for the individual items; there were no missing data at pre-BMT baseline.

Table 3 displays open responses to the item, “In the past week, how many days have you missed work or cut down on usual activities due to this child’s health?” At 45 days after BMT, 68% of parents reported missing work or cutting down usual activities at least one day in the previous week. At 3 months this value was 61%, and at one year, it was 33%. This item was moderately correlated with the PARimpact scale score, with Spearman’s Rank correlation values ranging from 0.28-0.57 across time measurements.

**Table 3:** **Parent self-reported days of missed work/decreased usual activity**

**Number of days**	**Day 45 n(%)**	**3 months n(%)**	**6 months n(%)**	**12 months n(%)**
**0**	93 (32)	111 (40)	140 (55)	156 (68)
**1-3**	53 (18)	70 (25)	61 (24)	44 (20)
**4-7**	146 (50)	100 (36)	55(21)	29 (13)

### Psychometric properties

#### PCA and EFA

PCA indicated a single component with an Eigen value of 2.6; all other Eigen values were less than 1.0. This component, “PARimpact,” explained 66% of the variation in the PARimpact scale. In the EFA, factor loadings ranged from 0.60 to 0.88, and uniqueness was <0.20. At pre-BMT baseline Cronbach’s alpha was 0.83, and at follow-up time points Cronbach’s alpha ranged from 0.84 – 0.90.

#### Construct validity

The PARimpact and Parent Emotional Functioning scales were strongly correlated (−0.57 to −0.67) across time periods. The Pearson correlation for PARimpact and Global QOL ranged from −0.49 to −0.63 across time periods. General Health, which was hypothesized to be the least conceptually similar to PARimpact, was weakly or moderately correlated with PARimpact; Pearson’s correlation ranged from −0.18 to −0.45 across time periods (see Table 4 for correlation coefficients at all time measurements).

**Table 4:** **Correlation coefficients for PARimpact, Global QOL, and General Health**

	**Pre-BMT**	**Day 45**	**3 months**	**6 months**	**1 year**
Parent Emotional Functioning	−0.57	−0.66	−0.60	−0.67	−0.66
Parent Global QOL	−0.49	−0.50	−0.57	−0.58	−0.63
Parent General Health	−0.18	−0.25	−0.37	−0.31	−0.45

#### Discriminant validity: results of known groups comparisons

Among parents completing the 3-month assessment, 71 (37%) BMT patients had early BMT complications. Parents of these children had a PARimpact score that was an average of 11.1 points higher than parents whose children did not experience early complications (t = 3.75; p < 0.01). Among parents completing the 12-month assessment, 86 (37%) experienced late BMT complications; parents whose children experienced late BMT complications averaged 6.8 points higher (t = 1.91; p = 0.06) than parents whose children did not.

### Regression analysis

#### Unadjusted analysis

In the early model, none of the demographic variables met the criteria to be included in the adjusted model. Among the early complication clinical variables, aGVHD (p < 0.01), Bearman Toxicity Score (p < 0.01), and systemic infection (p < 0.01) met the criteria to be included in the adjusted model.

In the late model, parent sex (p = 0.05) was the only demographic variables to be included in the adjusted model. Among the late complication clinical variables, systemic infection (p < 0.01) was the only variable to be included in the adjusted model.

#### Adjusted multivariable analysis

Based on AIC, a compound symmetry correlation structure was selected for the final model. Results of the likelihood ratio test comparing the PMM to the repeated measures model indicated the presence of MNAR (Early: χ^2^ (5) =12.7, p = 0.03; Late: χ^2^ (3) = 16.7, p<0.01), therefore PMM estimates were used in the final models. Estimated coefficients from the final models are displayed in Table 5.

**Table 5:** **Estimated coefficients for adjusted multivariable pattern mixture models**

	**Estimate**	**SE**	**p-value**
**Early (start of BMT – 3 months)**			
Time	−0.07	0.02	**<0.01**
aGVHD ≥ grade 2	5.31	2.48	**0.03**
Bearman Toxicity	5.91	1.97	**<0.01**
Systemic infection	9.09	2.09	**<0.01**
**Late (after 3 months - 1 year)**			
Time	−0.02	0.01	**0.02**
Systemic infection	19.18	3.14	**<0.01**

In the Early model, parents of children who experienced ≥ grade 2 aGVHD had a mean PARimpact score that was 5 points higher than parents of children who did not (p = 0.03). Furthermore, PARimpact scores averaged nearly 6 points higher among parents of children who had Bearman Toxicity Score of “intermediate” or “poor” (p < 0.01); and scores averaged 9 points higher among parents of children who experienced a systemic infection in the first 3 months following BMT (p < 0.01).

In the Late model, parent sex did not meet the criteria to be retained in the final model (p = 0.15). Among parents of children who experienced a systemic infection, mean PARimpact scores were 19 points higher than parents of children who did not experience a systemic infection (p < 0.01).

## Discussion

This study introduced the Parent Impact Scale, a four-item scale of the CHRIs General parent-proxy version. A scree plot and Eigen values supported a single factor solution, and PCA indicated a single factor explained more than 60% of variation. Factor loadings and uniqueness were acceptable, and the PARimpact scale demonstrated a strong coefficient of internal consistency reliability (α = 0.83).

The PARimpact also demonstrated hypothesized convergent and known groups validity. Specifically, the scale was strongly associated with global QOL and parent emotional functioning, but not with general health scales. In known group comparisons higher PARimpact scores were noted among parents whose children experienced early and late BMT complications (p < 0.01 and p = 0.06, respectively).

The additional open-response item of the PARimpact, assessed in follow-up time points, reveals that a high percentage of parents did miss work and/or cut down on usual activities during the year following BMT. This study is among the first to describe parents’ work patterns and/or decrease of usual activities post transplant. By 3 months post BMT, more than 2/3 of parents took at least one day off of work and/or cut down on usual activities during the week prior to the assessment *due to the child’s health*; by one year post BMT, nearly 1/3 of parents had taken off at least one day of work – a finding that reflects the high role disruption and associated economic burden that BMT places on families. This is consistent with other studies that have found more than one year after BMT, families of children who had BMT experience a greater financial impact than families of children who underwent treatment for leukemia, but did not require BMT [13]; and that 5–10 years after BMT, families of children who underwent BMT reported more financial strain than families of children with oncologic diagnoses who did not undergo BMT [11].

PMMs, which accounted for MNAR, showed mean PARimpact scores were significantly increased with clinical BMT complications, such as moderate to severe GVHD, “intermediate” or “poor” end organ toxicity, and systemic infection, which is consistent with other findings that parent emotional functioning declined with BMT clinical complications [5]. Systemic infection was associated with significantly higher mean PARimpact scores in both early and late models (9 points and 19 points higher respectively), which may be an indication of the distress and disruption that can be associated with a life-threatening complication like systemic infection at any point in the recovery trajectory. A more complete understanding of the durability of parent vulnerability after BMT could lead to the development of interventions to decrease parent impact during and after BMT. In a retrospective study more than one year after BMT, parents indicated that education related to taking care of themselves would be helpful to parents during the BMT process [34].

### Strengths and limitations

This study represents eight years of research; it is strengthened by the power of the large sample. Although the majority of the sample was White/Non-Hispanic (70%), as is typical of studies in this clinical population, the study team was able to recruit more than 27% Non-White or Hispanic participants. Study participants also had high educational attainment and income. The longitudinal design strengthens study findings, as does the completeness of clinical outcomes data even when patient-reported outcomes were missed. Nevertheless, as would be expected in a longitudinal study in a critically ill population, some study participants were lost to follow-up, principally due to the child’s death or relapse. Rigorous study procedures were used to mitigate loss to follow-up for non-medical reasons, and PMMs were used to account for data MNAR.

Another important limitation of the current study is that all of the survey measures used to assess the convergent validity of the PARimpact were self-reported by parent participants. This can result in shared variance, which may inflate measures of construct validity. Related data on missed work were also self-reported. However, we used clinical variables to examine known group validity, which were not parent reported. Finally, it is possible that there are other variables that contribute to parent impact during a child’s BMT that were not included in the PARimpact scale.

## Conclusion

The promising psychometric properties of the PARimpact scale indicate that parent impact is a unidimensional construct with clinical relevance. Our findings that early and late BMT complications, such as systemic infection, significantly increase the impact of BMT on the caretaking parent indicate the need for BMT healthcare professionals to identify these events and help parents navigate the BMT course. At minimum, BMT healthcare professionals should be mindful of the additional burden on the parent that complications bring, and proactively link parents to resources to help them cope with the added impact. Further study is needed to test the validity of the PARimpact scale in other populations of caretaking parents of children with serious illnesses.

## Abbreviations

AIC: Akaike information criteria
BMT: Blood and marrow transplantation
CHRIs: Child Health Rating Inventories
EFA: Exploratory factor analysis
aGVHD: Acute graft versus host disease
cGVHD: Chronic graft versus host disease
HRQL: Health related quality of life
JTR: Journeys to Recovery
MAR: Missing at random
MNAR: Missing not at random
PARimpact: Parent Impact Scale
PCA: Principal component analysis
PMM: Pattern mixture models
QOL: Quality of Life
REML: Residual maximum likelihood
